# Crystal structure of poly[[{μ_2_-1,4-bis[(1*H*-imid­azol-1-yl)methyl]benzene}[μ_6_-5-(4-carboxylatophenoxy)isophthalato]-μ_3_-hydroxido-dicobalt(II)] 0.25-hydrate]

**DOI:** 10.1107/S1600536814022806

**Published:** 2014-10-29

**Authors:** Yaping Li, Dajun Sun, Julia Ming, Liying Han, Guanfang Su

**Affiliations:** a Department of Ophthalmology, The Second Hospital of Jilin University, 218 Ziqiang Street, Changchun, 130041, Jilin Province, People’s Republic of China; b Department of Vascular Surgery, The China–Japan Union Hospital of Jilin University, 126 Xiantai Street, Changchun, 130033, Jilin Province, People’s Republic of China; c St Erik’s Eye Hospital, Karolinska Institutet, Polhemsgatan 50, SE-112-82, Stockholm, Sweden; d Department of Gynaecology, The Second Hospital of Jilin University, 218 Ziqiang Street, Changchun, 130041, Jilin Province, People’s Republic of China

**Keywords:** crystal structure, Co^II^ complex, (3,8)-connected tfz-d topology

## Abstract

The title coordination polymer, {[Co_2_(C_15_H_7_O_7_)(OH)(C_14_H_14_N_4_)]·0.25H_2_O}_*n*_, was synthesized under hydro­thermal conditions. The asymmetric unit contains two Co^2+^ ions, one *L* ^3−^ anion originating from 5-(4-carb­oxy­phen­oxy)isophthalic acid (H_3_ *L*), one OH^−^ ligand, one 1,4-bis­[(1*H*-imidazol-l-yl)meth­yl]benzene (bix) ligand and one disordered lattice water mol­ecule (occupancy 0.25). The two Co^2+^ ions have different environments. One has an octa­hedral O_4_N_2_ coordin­ation sphere, defined by four O atoms from three carboxyl­ate groups and one OH^−^ ligand, and two N atoms from two symmetry-related bix ligands. The other has a trigonal-bipyramidal O_5_ coordination sphere resulting from three carboxyl­ate groups and two OH^−^ ligands. The dihedral angles between the two benzene rings in the *L* ^3−^ ligand and between the benzene ring and the two imidazole rings in the bix ligand are 67.05 (15), 75.27 (17) and 82.05 (17)°, respectively. Four neighbouring Co^2+^ ions are linked by six carboxyl­ate groups and two *μ*_3_-OH ligands, forming a butterfly-shaped secondary building unit (SBU). These SBUs are connected by *L* ^3−^ anions into layers parallel to (1-10). Adjacent layers are cross-linked by the bix ligands, forming a three-dimensional framework that has a bimodal (3,8)-connected tfz-d topology. The disordered lattice water mol­ecule is located in the voids of the framework and has O⋯O and O⋯N contacts of 2.81 (2) and 2.95 (2) Å, suggesting medium-strength hydrogen bonds. The title compound may be a good candidate for artificial eye lenses.

## Related literature   

For general background to the properties and applications of compounds with metal-organic framework structures (MOFs), see: Batten & Robson (1998 ▶); Farrusseng *et al.* (2009 ▶); Iremonger *et al.* (2013 ▶); Kreno *et al.* (2012 ▶); Kurmoo (2009 ▶); Song *et al.* (2013 ▶); Su *et al.* (2012 ▶); Wong *et al.* (2006 ▶). For topological analysis of crystal structures, see: Blatov *et al.* (2010 ▶).
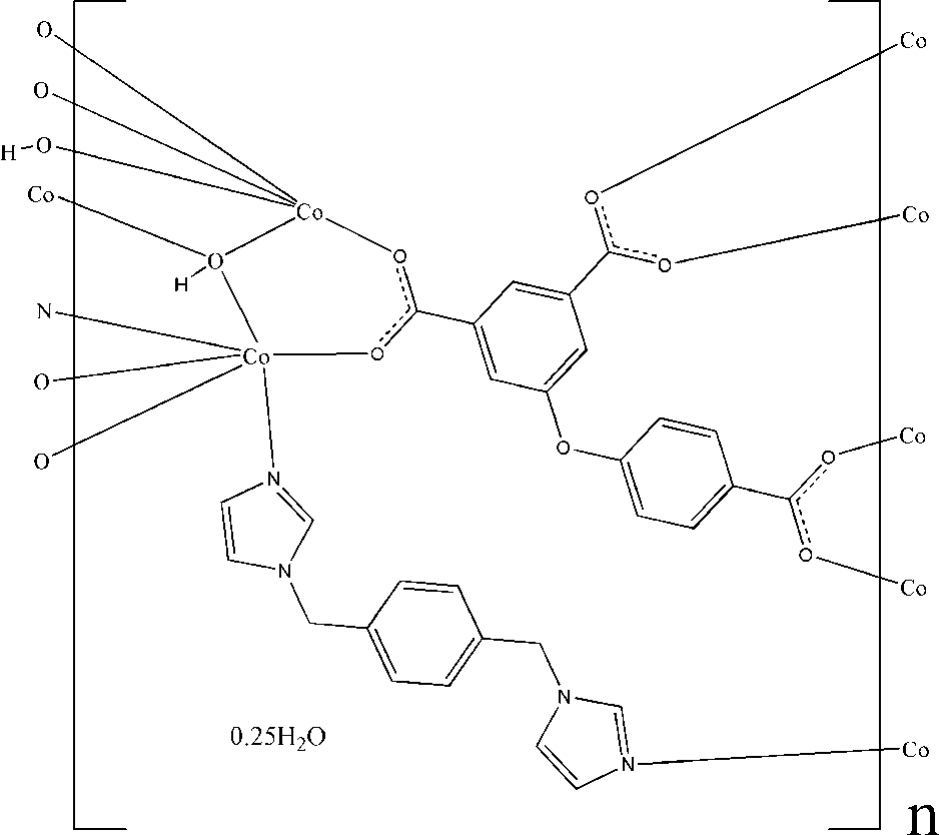


## Experimental   

### Crystal data  

[Co_2_(C_15_H_7_O_7_)(OH)(C_14_H_14_N_4_)]·0.25H_2_O
*M* *_r_* = 676.87
Triclinic, 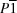

*a* = 10.7381 (6) Å
*b* = 10.7477 (6) Å
*c* = 13.5585 (12) Å
α = 95.596 (1)°
β = 91.497 (1)°
γ = 118.728 (1)°
*V* = 1360.85 (16) Å^3^
*Z* = 2
Mo *K*α radiation
μ = 1.28 mm^−1^
*T* = 173 K
0.19 × 0.16 × 0.15 mm

### Data collection  

Bruker APEXII CCD diffractometer
Absorption correction: multi-scan (*SADABS*; Bruker, 2010 ▶) *T*_min_ = 0.793, *T*_max_ = 0.831
7626 measured reflections
5314 independent reflections
4359 reflections with *I* > 2σ(*I*)
*R*_int_ = 0.024

### Refinement  

*R*[*F* ^2^ > 2σ(*F* ^2^)] = 0.037
*wR*(*F* ^2^) = 0.086
*S* = 1.04
5314 reflections
396 parameters
1 restraint
H atoms treated by a mixture of independent and constrained refinement
Δρ_max_ = 0.37 e Å^−3^
Δρ_min_ = −0.37 e Å^−3^

### 

Data collection: *APEX2* (Bruker, 2010 ▶); cell refinement: *SAINT* (Bruker, 2010 ▶); data reduction: *SAINT* (Bruker, 2010 ▶); program(s) used to solve structure: *SHELXS97* (Sheldrick, 2008 ▶); program(s) used to refine structure: *SHELXL97* (Sheldrick, 2008 ▶); molecular graphics: *XP* in *SHELXTL* (Sheldrick, 2008 ▶) and *DIAMOND* (Brandenburg & Putz, 2010 ▶); software used to prepare material for publication: *publCIF* (Westrip, 2010 ▶).

## Supplementary Material

Crystal structure: contains datablock(s) I, New_Global_Publ_Block. DOI: 10.1107/S1600536814022806/wm5040sup1.cif

**Fig. 1.**
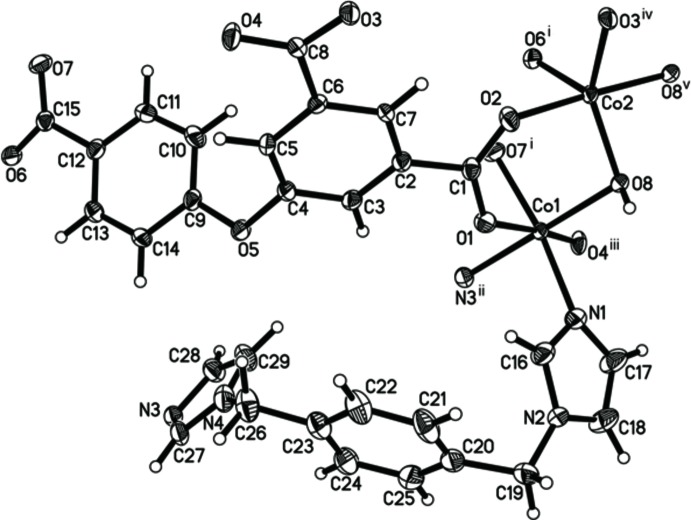
The extended asymmetric unit of the title compound. Displacement ellipsoids are drawn at the 50% probability level. The disordered lattice water molecule has been omitted for clarity. [Symmetry codes: i) 2 - x, 2 - y, 1 - z; ii) 2 - x, 1 - y, 1 - z; iii) -1 + x, -1 + y, z; iv) 2 - x, 2 - y, -z; v) 1 - x, 1 - y, z.]

**Fig. 2.**
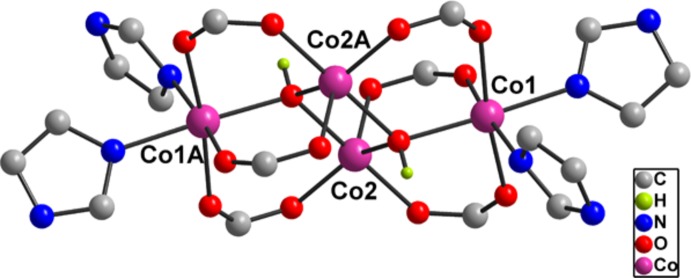
The tetranuclear SBU in the structure of the title compound. [Symmetry code: A) 1 - x,1 - y,-z.]

Structure factors: contains datablock(s) I. DOI: 10.1107/S1600536814022806/wm5040Isup2.hkl

x y z x y z x y z x y z x y z . DOI: 10.1107/S1600536814022806/wm5040fig1.tif
The extended asymmetric unit of the title compound. Displacement ellipsoids are drawn at the 50% probability level. The disordered lattice water mol­ecule has been omitted for clarity. [Symmetry codes: i) 2 − *x*, 2 − *y*, 1 − *z*; ii) 2 − *x*, 1 − *y*, 1 − *z*; iii) −1 + *x*, −1 + *y*, *z*; iv) 2 − *x*, 2 − *y*, −*z*; v) 1 − *x*, 1 − *y*, *z*.]

x y z . DOI: 10.1107/S1600536814022806/wm5040fig2.tif
The tetra­nuclear SBU in the structure of the title compound. [Symmetry code: A) 1 − *x*,1 − *y*,-*z*.]

**Fig. 3.**
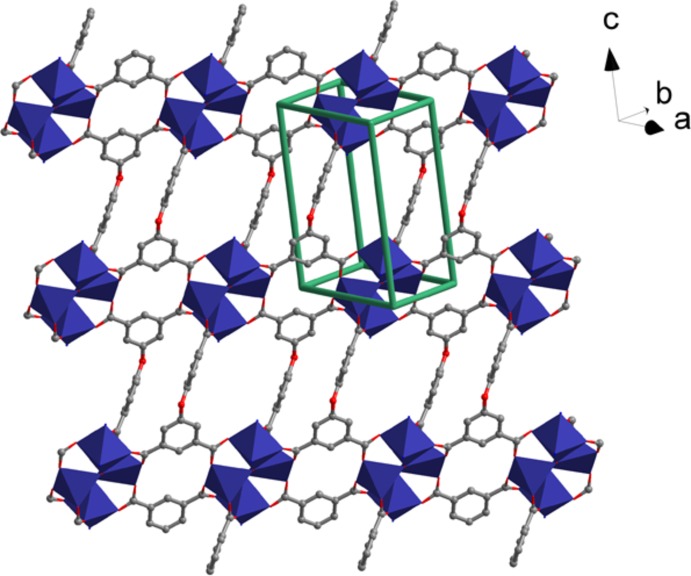
View of the layered network formed by the SBUs and the L3- anions.

L 3− . DOI: 10.1107/S1600536814022806/wm5040fig3.tif
View of the layered network formed by the SBUs and the *L* ^3−^ anions.

CCDC reference: 1029647

Additional supporting information:  crystallographic information; 3D view; checkCIF report

## supplementary crystallographic information

### S1. Synthesis and crystallization

A mixture of cobalt acetate tetra­hydrate (0.0249 g, 0.1 mmol), 5-(4-carb­oxy­phen­oxy)­isophthalic acid (H_3_*L*, 0.0151 g, 0.05 mmol), 1,4-bis­[(1*H*-imidazol-l-yl)methyl]­benzene (bix) (0.0118 g, 0.05 mmol), water (6 ml) and NaOH (aq, 0.1 molar, 2 ml) was placed in a 20 ml PTFE-lined stainless steel vessel under autogenous pressure, heated at a 413 K for 5 days, and allowed to cool down to room temperature during 30 h. The obtained crystals were collected, washed with water and ethanol, and dried under ambient conditions with a yield of 17% based on cobalt acetate.

### S2. Refinement

H atoms attached to C atoms were placed in geometrically idealized positions and constrained to ride on their parent atoms with a C—H distance of 0.95 Å (aromatic) and 0.98 Å (methyl­ene), and with *U*_iso_(H) = 1.2*U*_eq_(C). The H atom of the hy­droxy group was located from a difference Fourier map and was refined with a distance restraint of 0.85 (2) Å. Additional electron density was found that was assigned to a lattice water molecule. Refinement of its occupancy revealed a considerable under-occupation that was fixed at 0.25 for the final refinement. H atoms of this molecule were not considered in the final model.

### Crystal data

**Table:**

[Co_2_(C_15_H_7_O_7_)(OH)(C_14_H_14_N_4_)]·0.25H_2_O	*V* = 1360.85 (16) Å^3^
*M**_r_* = 676.87	*Z* = 2
Triclinic, *P*1	*F*(000) = 688
Hall symbol: -P 1	*D*_x_ = 1.651 Mg m^−^^3^
*a* = 10.7381 (6) Å	Mo *K*α radiation, λ = 0.71073 Å
*b* = 10.7477 (6) Å	µ = 1.28 mm^−^^1^
*c* = 13.5585 (12) Å	*T* = 173 K
α = 95.596 (1)°	Block, red
β = 91.497 (1)°	0.19 × 0.16 × 0.15 mm
γ = 118.728 (1)°	

### Data collection

**Table:**

Bruker APEXII CCD diffractometer	5314 independent reflections
Radiation source: fine-focus sealed tube	4359 reflections with *I* > 2σ(*I*)
Graphite monochromator	*R*_int_ = 0.024
φ and ω scans	θ_max_ = 26.1°, θ_min_ = 2.2°
Absorption correction: multi-scan (*SADABS*; Bruker, 2010)	*h* = −13→7
*T*_min_ = 0.793, *T*_max_ = 0.831	*k* = −12→13
7626 measured reflections	*l* = −16→15

### Refinement

**Table:**

Refinement on *F*^2^	Primary atom site location: structure-invariant direct methods
Least-squares matrix: full	Secondary atom site location: difference Fourier map
*R*[*F*^2^ > 2σ(*F*^2^)] = 0.037	Hydrogen site location: inferred from neighbouring sites
*wR*(*F*^2^) = 0.086	H atoms treated by a mixture of independent and constrained refinement
*S* = 1.04	*w* = 1/[σ^2^(*F*_o_^2^) + (0.038*P*)^2^ + 0.1129*P*] where *P* = (*F*_o_^2^ + 2*F*_c_^2^)/3
5314 reflections	(Δ/σ)_max_ = 0.001
396 parameters	Δρ_max_ = 0.37 e Å^−^^3^
1 restraint	Δρ_min_ = −0.37 e Å^−^^3^

### Special details

**Table:**

Geometry. All e.s.d.'s (except the e.s.d. in the dihedral angle between two l.s. planes) are estimated using the full covariance matrix. The cell e.s.d.'s are taken into account individually in the estimation of e.s.d.'s in distances, angles and torsion angles; correlations between e.s.d.'s in cell parameters are only used when they are defined by crystal symmetry. An approximate (isotropic) treatment of cell e.s.d.'s is used for estimating e.s.d.'s involving l.s. planes.
Refinement. Refinement of *F*^2^ against ALL reflections. The weighted *R*-factor *wR* and goodness of fit *S* are based on *F*^2^, conventional *R*-factors *R* are based on *F*, with *F* set to zero for negative *F*^2^. The threshold expression of *F*^2^ > σ(*F*^2^) is used only for calculating *R*-factors(gt) *etc*. and is not relevant to the choice of reflections for refinement. *R*-factors based on *F*^2^ are statistically about twice as large as those based on *F*, and *R*- factors based on ALL data will be even larger.

### Fractional atomic coordinates and isotropic or equivalent isotropic displacement parameters (Å^2^)

**Table:**

	*x*	*y*	*z*	*U*_iso_*/*U*_eq_	Occ. (<1)
C4	1.2218 (3)	0.9155 (3)	0.33134 (18)	0.0184 (6)	
C5	1.3041 (3)	1.0414 (3)	0.29083 (18)	0.0174 (6)	
H5	1.3904	1.1145	0.3255	0.021*	
C6	1.2583 (3)	1.0586 (3)	0.19865 (18)	0.0142 (5)	
C7	1.1319 (3)	0.9514 (3)	0.14908 (18)	0.0149 (5)	
H7	1.0997	0.9645	0.0870	0.018*	
C8	1.3462 (3)	1.1946 (3)	0.15375 (18)	0.0159 (5)	
C9	1.2867 (3)	0.9886 (3)	0.50570 (18)	0.0189 (6)	
C10	1.2320 (3)	1.0822 (3)	0.5104 (2)	0.0260 (6)	
H10	1.1758	1.0828	0.4553	0.031*	
C11	1.2605 (3)	1.1752 (3)	0.5968 (2)	0.0235 (6)	
H11	1.2248	1.2409	0.5998	0.028*	
C12	1.3400 (3)	1.1742 (3)	0.67892 (19)	0.0191 (6)	
C13	1.3897 (3)	1.0762 (3)	0.67393 (19)	0.0217 (6)	
H13	1.4413	1.0720	0.7303	0.026*	
C14	1.3646 (3)	0.9841 (3)	0.58730 (19)	0.0217 (6)	
H14	1.4005	0.9186	0.5839	0.026*	
C15	1.3742 (3)	1.2825 (3)	0.76871 (19)	0.0186 (6)	
C16	0.8332 (3)	0.2942 (3)	0.1162 (2)	0.0271 (7)	
H16	0.9160	0.3856	0.1245	0.033*	
C17	0.6168 (3)	0.1286 (3)	0.0961 (2)	0.0338 (7)	
H17	0.5157	0.0803	0.0878	0.041*	
C18	0.6975 (3)	0.0639 (3)	0.0971 (2)	0.0339 (7)	
H18	0.6645	−0.0361	0.0897	0.041*	
C19	0.9626 (3)	0.1515 (3)	0.1249 (2)	0.0323 (7)	
H19A	1.0080	0.1586	0.0616	0.039*	
H19B	0.9334	0.0554	0.1444	0.039*	
C20	1.0678 (3)	0.2645 (3)	0.2043 (2)	0.0287 (7)	
C21	1.1660 (3)	0.3964 (4)	0.1793 (2)	0.0356 (8)	
H21	1.1718	0.4114	0.1113	0.043*	
C22	1.2558 (3)	0.5068 (4)	0.2509 (2)	0.0343 (8)	
H22	1.3203	0.5975	0.2321	0.041*	
C23	1.2520 (3)	0.4855 (3)	0.3500 (2)	0.0281 (7)	
C24	1.1572 (3)	0.3524 (3)	0.3759 (2)	0.0342 (8)	
H24	1.1557	0.3360	0.4435	0.041*	
C25	1.0645 (3)	0.2427 (3)	0.3038 (2)	0.0345 (7)	
H25	0.9986	0.1526	0.3226	0.041*	
C26	1.3528 (3)	0.6094 (3)	0.4265 (2)	0.0342 (8)	
H26A	1.4452	0.6102	0.4324	0.041*	
H26B	1.3701	0.7002	0.4029	0.041*	
C27	1.3559 (3)	0.5860 (3)	0.6085 (2)	0.0255 (6)	
H27	1.4409	0.5794	0.6127	0.031*	
C28	1.1671 (3)	0.5928 (3)	0.6463 (2)	0.0288 (7)	
H28	1.0927	0.5913	0.6835	0.035*	
C29	1.1776 (3)	0.6075 (3)	0.5482 (2)	0.0315 (7)	
H29	1.1141	0.6190	0.5047	0.038*	
N3	1.2798 (2)	0.5803 (2)	0.68379 (16)	0.0221 (5)	
N4	1.2985 (3)	0.6023 (3)	0.52460 (17)	0.0277 (6)	
O3	1.30271 (18)	1.19794 (18)	0.06670 (12)	0.0196 (4)	
O4	1.45267 (18)	1.29275 (18)	0.20484 (13)	0.0215 (4)	
O5	1.2654 (2)	0.89221 (18)	0.42257 (12)	0.0223 (4)	
O6	1.4105 (2)	1.25737 (19)	0.85136 (13)	0.0226 (4)	
O7	1.3637 (2)	1.39052 (19)	0.75613 (13)	0.0248 (4)	
O8	0.58483 (19)	0.45012 (18)	0.02893 (13)	0.0166 (4)	
O1W	1.333 (2)	0.746 (2)	0.1092 (16)	0.165 (8)*	0.25
H8O	0.632 (3)	0.422 (3)	−0.0057 (19)	0.030 (9)*	
C1	0.9122 (3)	0.7102 (3)	0.13668 (18)	0.0178 (6)	
C2	1.0516 (3)	0.8247 (3)	0.18914 (18)	0.0164 (5)	
C3	1.0979 (3)	0.8066 (3)	0.28083 (19)	0.0186 (6)	
H3	1.0445	0.7200	0.3085	0.022*	
N1	0.7019 (2)	0.2740 (2)	0.10884 (17)	0.0235 (5)	
N2	0.8355 (2)	0.1701 (2)	0.11079 (16)	0.0225 (5)	
O1	0.86795 (18)	0.58516 (18)	0.15727 (14)	0.0230 (4)	
O2	0.84879 (19)	0.74906 (19)	0.07745 (13)	0.0236 (4)	
Co1	0.65561 (4)	0.43648 (3)	0.16976 (2)	0.01604 (10)	
Co2	0.63498 (4)	0.65449 (3)	0.02558 (2)	0.01564 (10)	

### Atomic displacement parameters (Å^2^)

**Table:**

	*U*^11^	*U*^22^	*U*^33^	*U*^12^	*U*^13^	*U*^23^
C4	0.0237 (14)	0.0168 (13)	0.0139 (13)	0.0096 (12)	−0.0039 (11)	0.0018 (11)
C5	0.0176 (13)	0.0149 (13)	0.0162 (13)	0.0058 (11)	−0.0041 (11)	−0.0010 (10)
C6	0.0146 (13)	0.0114 (12)	0.0153 (13)	0.0053 (10)	0.0013 (10)	0.0016 (10)
C7	0.0168 (13)	0.0147 (13)	0.0134 (12)	0.0080 (11)	−0.0032 (10)	0.0016 (10)
C8	0.0151 (13)	0.0157 (13)	0.0169 (13)	0.0077 (11)	−0.0019 (11)	0.0019 (11)
C9	0.0240 (14)	0.0160 (13)	0.0120 (13)	0.0065 (12)	−0.0025 (11)	0.0009 (10)
C10	0.0348 (17)	0.0297 (16)	0.0174 (14)	0.0195 (14)	−0.0073 (12)	0.0004 (12)
C11	0.0288 (16)	0.0224 (15)	0.0230 (14)	0.0159 (13)	−0.0035 (12)	0.0021 (12)
C12	0.0225 (14)	0.0146 (13)	0.0156 (13)	0.0054 (11)	−0.0009 (11)	0.0024 (11)
C13	0.0248 (15)	0.0229 (15)	0.0165 (13)	0.0111 (12)	−0.0050 (11)	0.0027 (11)
C14	0.0299 (16)	0.0211 (14)	0.0171 (14)	0.0155 (13)	−0.0063 (12)	0.0006 (11)
C15	0.0163 (13)	0.0167 (14)	0.0185 (14)	0.0045 (11)	0.0002 (11)	0.0035 (11)
C16	0.0248 (16)	0.0171 (14)	0.0359 (17)	0.0082 (13)	−0.0020 (13)	−0.0008 (13)
C17	0.0245 (16)	0.0193 (15)	0.051 (2)	0.0075 (13)	0.0014 (14)	−0.0042 (14)
C18	0.0354 (18)	0.0161 (15)	0.047 (2)	0.0097 (14)	0.0055 (15)	0.0023 (14)
C19	0.0388 (18)	0.0387 (18)	0.0306 (17)	0.0297 (16)	−0.0061 (14)	−0.0051 (14)
C20	0.0317 (17)	0.0363 (18)	0.0267 (16)	0.0252 (15)	−0.0032 (13)	−0.0060 (13)
C21	0.0322 (18)	0.060 (2)	0.0190 (15)	0.0266 (17)	0.0005 (13)	0.0028 (15)
C22	0.0253 (17)	0.044 (2)	0.0289 (17)	0.0129 (15)	0.0007 (13)	0.0078 (15)
C23	0.0265 (16)	0.0365 (18)	0.0230 (15)	0.0165 (14)	0.0007 (12)	0.0045 (13)
C24	0.046 (2)	0.0370 (18)	0.0225 (16)	0.0226 (16)	−0.0071 (14)	0.0034 (14)
C25	0.044 (2)	0.0308 (17)	0.0320 (17)	0.0221 (16)	−0.0051 (15)	0.0015 (14)
C26	0.0279 (17)	0.0392 (19)	0.0248 (16)	0.0075 (15)	−0.0015 (13)	0.0070 (14)
C27	0.0231 (15)	0.0245 (15)	0.0243 (15)	0.0087 (13)	−0.0089 (12)	0.0015 (12)
C28	0.0274 (16)	0.0282 (16)	0.0300 (16)	0.0126 (14)	−0.0033 (13)	0.0064 (13)
C29	0.0261 (16)	0.0342 (18)	0.0313 (17)	0.0121 (14)	−0.0063 (13)	0.0083 (14)
N3	0.0227 (13)	0.0186 (12)	0.0223 (12)	0.0084 (10)	−0.0047 (10)	0.0019 (10)
N4	0.0277 (14)	0.0245 (13)	0.0230 (13)	0.0066 (11)	−0.0042 (10)	0.0035 (10)
O3	0.0197 (10)	0.0154 (9)	0.0169 (9)	0.0028 (8)	−0.0040 (8)	0.0057 (8)
O4	0.0186 (10)	0.0165 (10)	0.0182 (9)	0.0000 (8)	−0.0043 (8)	0.0018 (8)
O5	0.0359 (12)	0.0168 (10)	0.0123 (9)	0.0119 (9)	−0.0070 (8)	0.0007 (7)
O6	0.0324 (11)	0.0194 (10)	0.0159 (9)	0.0133 (9)	−0.0044 (8)	−0.0009 (8)
O7	0.0352 (12)	0.0159 (10)	0.0236 (10)	0.0131 (9)	−0.0012 (9)	0.0006 (8)
O8	0.0170 (10)	0.0111 (9)	0.0185 (9)	0.0046 (8)	−0.0016 (8)	0.0006 (7)
C1	0.0170 (13)	0.0163 (14)	0.0152 (13)	0.0042 (11)	0.0010 (11)	0.0027 (11)
C2	0.0172 (13)	0.0141 (13)	0.0160 (13)	0.0062 (11)	0.0000 (11)	0.0018 (10)
C3	0.0218 (14)	0.0137 (13)	0.0182 (13)	0.0069 (11)	−0.0001 (11)	0.0033 (11)
N1	0.0225 (12)	0.0168 (12)	0.0292 (13)	0.0086 (10)	−0.0038 (10)	0.0005 (10)
N2	0.0278 (13)	0.0208 (12)	0.0219 (12)	0.0155 (11)	−0.0028 (10)	−0.0028 (10)
O1	0.0160 (10)	0.0139 (9)	0.0337 (11)	0.0030 (8)	−0.0038 (8)	0.0049 (8)
O2	0.0169 (10)	0.0174 (10)	0.0257 (10)	−0.0009 (8)	−0.0063 (8)	0.0079 (8)
Co1	0.01564 (19)	0.01004 (18)	0.01836 (19)	0.00323 (15)	−0.00388 (14)	0.00201 (14)
Co2	0.01722 (19)	0.01009 (18)	0.01444 (18)	0.00264 (15)	−0.00307 (14)	0.00217 (14)

### Geometric parameters (Å, º)

**Table:**

C4—C3	1.381 (4)	C22—C23	1.383 (4)
C4—C5	1.391 (3)	C22—H22	0.9500
C4—O5	1.398 (3)	C23—C24	1.386 (4)
C5—C6	1.393 (3)	C23—C26	1.519 (4)
C5—H5	0.9500	C24—C25	1.390 (4)
C6—C7	1.386 (3)	C24—H24	0.9500
C6—C8	1.508 (3)	C25—H25	0.9500
C7—C2	1.390 (3)	C26—N4	1.460 (4)
C7—H7	0.9500	C26—H26A	0.9900
C8—O4	1.248 (3)	C26—H26B	0.9900
C8—O3	1.268 (3)	C27—N3	1.312 (4)
C9—C10	1.385 (4)	C27—N4	1.346 (3)
C9—C14	1.388 (3)	C27—H27	0.9500
C9—O5	1.388 (3)	C28—C29	1.357 (4)
C10—C11	1.386 (4)	C28—N3	1.371 (3)
C10—H10	0.9500	C28—H28	0.9500
C11—C12	1.390 (3)	C29—N4	1.370 (4)
C11—H11	0.9500	C29—H29	0.9500
C12—C13	1.388 (4)	N3—Co1^i^	2.139 (2)
C12—C15	1.504 (4)	O3—Co2^ii^	1.9848 (16)
C13—C14	1.390 (4)	O4—Co1^iii^	2.0791 (17)
C13—H13	0.9500	O6—Co2^iv^	2.0247 (18)
C14—H14	0.9500	O7—Co1^iv^	2.1279 (19)
C15—O7	1.246 (3)	O8—Co2	2.0000 (17)
C15—O6	1.272 (3)	O8—Co1	2.0811 (17)
C16—N1	1.318 (4)	O8—Co2^v^	2.1395 (18)
C16—N2	1.341 (3)	O8—H8O	0.839 (17)
C16—H16	0.9500	C1—O1	1.256 (3)
C17—C18	1.347 (4)	C1—O2	1.262 (3)
C17—N1	1.369 (4)	C1—C2	1.505 (3)
C17—H17	0.9500	C2—C3	1.392 (3)
C18—N2	1.360 (4)	C3—H3	0.9500
C18—H18	0.9500	N1—Co1	2.134 (2)
C19—N2	1.482 (3)	O1—Co1	2.0860 (17)
C19—C20	1.508 (4)	O2—Co2	2.0813 (18)
C19—H19A	0.9900	Co1—O4^vi^	2.0791 (17)
C19—H19B	0.9900	Co1—O7^iv^	2.1279 (19)
C20—C21	1.383 (4)	Co1—N3^i^	2.139 (2)
C20—C25	1.390 (4)	Co2—O3^ii^	1.9848 (16)
C21—C22	1.381 (4)	Co2—O6^iv^	2.0247 (18)
C21—H21	0.9500	Co2—O8^v^	2.1395 (18)
			
C3—C4—C5	121.2 (2)	N4—C26—H26B	108.9
C3—C4—O5	117.7 (2)	C23—C26—H26B	108.9
C5—C4—O5	120.9 (2)	H26A—C26—H26B	107.8
C4—C5—C6	119.0 (2)	N3—C27—N4	111.6 (3)
C4—C5—H5	120.5	N3—C27—H27	124.2
C6—C5—H5	120.5	N4—C27—H27	124.2
C7—C6—C5	119.9 (2)	C29—C28—N3	109.8 (3)
C7—C6—C8	120.4 (2)	C29—C28—H28	125.1
C5—C6—C8	119.7 (2)	N3—C28—H28	125.1
C6—C7—C2	120.7 (2)	C28—C29—N4	106.0 (3)
C6—C7—H7	119.6	C28—C29—H29	127.0
C2—C7—H7	119.6	N4—C29—H29	127.0
O4—C8—O3	126.2 (2)	C27—N3—C28	105.6 (2)
O4—C8—C6	118.0 (2)	C27—N3—Co1^i^	121.73 (19)
O3—C8—C6	115.9 (2)	C28—N3—Co1^i^	132.6 (2)
C10—C9—C14	120.6 (2)	C27—N4—C29	107.0 (2)
C10—C9—O5	123.5 (2)	C27—N4—C26	126.5 (3)
C14—C9—O5	115.8 (2)	C29—N4—C26	126.5 (2)
C9—C10—C11	119.0 (2)	C8—O3—Co2^ii^	134.31 (16)
C9—C10—H10	120.5	C8—O4—Co1^iii^	132.65 (17)
C11—C10—H10	120.5	C9—O5—C4	118.20 (19)
C10—C11—C12	121.4 (3)	C15—O6—Co2^iv^	115.77 (17)
C10—C11—H11	119.3	C15—O7—Co1^iv^	144.04 (18)
C12—C11—H11	119.3	Co2—O8—Co1	106.75 (8)
C13—C12—C11	118.7 (2)	Co2—O8—Co2^v^	100.27 (8)
C13—C12—C15	122.1 (2)	Co1—O8—Co2^v^	123.61 (8)
C11—C12—C15	119.2 (2)	Co2—O8—H8O	116 (2)
C12—C13—C14	120.7 (2)	Co1—O8—H8O	99 (2)
C12—C13—H13	119.7	Co2^v^—O8—H8O	112 (2)
C14—C13—H13	119.7	O1—C1—O2	125.9 (2)
C9—C14—C13	119.5 (3)	O1—C1—C2	117.1 (2)
C9—C14—H14	120.2	O2—C1—C2	117.0 (2)
C13—C14—H14	120.2	C7—C2—C3	119.5 (2)
O7—C15—O6	124.9 (2)	C7—C2—C1	120.6 (2)
O7—C15—C12	116.9 (2)	C3—C2—C1	119.8 (2)
O6—C15—C12	118.2 (2)	C4—C3—C2	119.6 (2)
N1—C16—N2	111.8 (2)	C4—C3—H3	120.2
N1—C16—H16	124.1	C2—C3—H3	120.2
N2—C16—H16	124.1	C16—N1—C17	104.8 (2)
C18—C17—N1	110.1 (3)	C16—N1—Co1	120.66 (19)
C18—C17—H17	125.0	C17—N1—Co1	129.3 (2)
N1—C17—H17	125.0	C16—N2—C18	106.8 (2)
C17—C18—N2	106.5 (2)	C16—N2—C19	126.8 (2)
C17—C18—H18	126.8	C18—N2—C19	126.2 (2)
N2—C18—H18	126.8	C1—O1—Co1	125.59 (17)
N2—C19—C20	110.2 (2)	C1—O2—Co2	130.90 (16)
N2—C19—H19A	109.6	O4^vi^—Co1—O8	93.75 (7)
C20—C19—H19A	109.6	O4^vi^—Co1—O1	171.43 (7)
N2—C19—H19B	109.6	O8—Co1—O1	94.52 (7)
C20—C19—H19B	109.6	O4^vi^—Co1—O7^iv^	90.30 (7)
H19A—C19—H19B	108.1	O8—Co1—O7^iv^	95.87 (7)
C21—C20—C25	118.4 (3)	O1—Co1—O7^iv^	86.61 (7)
C21—C20—C19	119.7 (3)	O4^vi^—Co1—N1	94.34 (8)
C25—C20—C19	121.7 (3)	O8—Co1—N1	90.45 (8)
C22—C21—C20	121.5 (3)	O1—Co1—N1	87.85 (8)
C22—C21—H21	119.2	O7^iv^—Co1—N1	171.91 (8)
C20—C21—H21	119.2	O4^vi^—Co1—N3^i^	84.20 (8)
C21—C22—C23	120.0 (3)	O8—Co1—N3^i^	177.78 (8)
C21—C22—H22	120.0	O1—Co1—N3^i^	87.50 (8)
C23—C22—H22	120.0	O7^iv^—Co1—N3^i^	83.32 (8)
C22—C23—C24	119.1 (3)	N1—Co1—N3^i^	90.55 (9)
C22—C23—C26	118.5 (3)	O3^ii^—Co2—O8	140.83 (8)
C24—C23—C26	122.5 (3)	O3^ii^—Co2—O6^iv^	104.88 (7)
C23—C24—C25	120.7 (3)	O8—Co2—O6^iv^	113.82 (7)
C23—C24—H24	119.7	O3^ii^—Co2—O2	86.27 (7)
C25—C24—H24	119.7	O8—Co2—O2	98.24 (7)
C20—C25—C24	120.2 (3)	O6^iv^—Co2—O2	91.54 (8)
C20—C25—H25	119.9	O3^ii^—Co2—O8^v^	94.91 (7)
C24—C25—H25	119.9	O8—Co2—O8^v^	79.73 (8)
N4—C26—C23	113.2 (2)	O6^iv^—Co2—O8^v^	89.87 (7)
N4—C26—H26A	108.9	O2—Co2—O8^v^	177.87 (7)
C23—C26—H26A	108.9		
			
C3—C4—C5—C6	1.6 (4)	C6—C7—C2—C3	1.0 (4)
O5—C4—C5—C6	178.5 (2)	C6—C7—C2—C1	178.1 (2)
C4—C5—C6—C7	0.3 (4)	O1—C1—C2—C7	158.0 (2)
C4—C5—C6—C8	179.9 (2)	O2—C1—C2—C7	−23.4 (4)
C5—C6—C7—C2	−1.6 (4)	O1—C1—C2—C3	−24.9 (4)
C8—C6—C7—C2	178.8 (2)	O2—C1—C2—C3	153.7 (2)
C7—C6—C8—O4	174.1 (2)	C5—C4—C3—C2	−2.2 (4)
C5—C6—C8—O4	−5.5 (4)	O5—C4—C3—C2	−179.2 (2)
C7—C6—C8—O3	−5.3 (4)	C7—C2—C3—C4	0.9 (4)
C5—C6—C8—O3	175.1 (2)	C1—C2—C3—C4	−176.3 (2)
C14—C9—C10—C11	2.3 (4)	N2—C16—N1—C17	−1.2 (3)
O5—C9—C10—C11	−179.5 (2)	N2—C16—N1—Co1	155.63 (18)
C9—C10—C11—C12	−1.3 (4)	C18—C17—N1—C16	0.7 (4)
C10—C11—C12—C13	−0.9 (4)	C18—C17—N1—Co1	−153.4 (2)
C10—C11—C12—C15	176.8 (3)	N1—C16—N2—C18	1.2 (3)
C11—C12—C13—C14	2.2 (4)	N1—C16—N2—C19	−174.3 (3)
C15—C12—C13—C14	−175.4 (2)	C17—C18—N2—C16	−0.8 (3)
C10—C9—C14—C13	−1.0 (4)	C17—C18—N2—C19	174.8 (3)
O5—C9—C14—C13	−179.4 (2)	C20—C19—N2—C16	36.0 (4)
C12—C13—C14—C9	−1.3 (4)	C20—C19—N2—C18	−138.8 (3)
C13—C12—C15—O7	158.7 (3)	O2—C1—O1—Co1	−36.0 (4)
C11—C12—C15—O7	−18.9 (4)	C2—C1—O1—Co1	142.50 (19)
C13—C12—C15—O6	−21.7 (4)	O1—C1—O2—Co2	21.4 (4)
C11—C12—C15—O6	160.7 (2)	C2—C1—O2—Co2	−157.07 (18)
N1—C17—C18—N2	0.1 (4)	Co2—O8—Co1—O4^vi^	119.02 (9)
N2—C19—C20—C21	−86.0 (3)	Co2^v^—O8—Co1—O4^vi^	3.95 (11)
N2—C19—C20—C25	90.5 (3)	Co2—O8—Co1—O1	−58.72 (9)
C25—C20—C21—C22	−2.4 (5)	Co2^v^—O8—Co1—O1	−173.79 (10)
C19—C20—C21—C22	174.3 (3)	Co2—O8—Co1—O7^iv^	28.33 (9)
C20—C21—C22—C23	2.0 (5)	Co2^v^—O8—Co1—O7^iv^	−86.74 (11)
C21—C22—C23—C24	0.1 (5)	Co2—O8—Co1—N1	−146.60 (9)
C21—C22—C23—C26	179.8 (3)	Co2^v^—O8—Co1—N1	98.33 (11)
C22—C23—C24—C25	−1.8 (5)	Co2—O8—Co1—N3^i^	97 (2)
C26—C23—C24—C25	178.5 (3)	Co2^v^—O8—Co1—N3^i^	−18 (2)
C21—C20—C25—C24	0.7 (5)	C1—O1—Co1—O4^vi^	−109.8 (5)
C19—C20—C25—C24	−175.9 (3)	C1—O1—Co1—O8	54.9 (2)
C23—C24—C25—C20	1.4 (5)	C1—O1—Co1—O7^iv^	−40.8 (2)
C22—C23—C26—N4	152.3 (3)	C1—O1—Co1—N1	145.2 (2)
C24—C23—C26—N4	−28.0 (4)	C1—O1—Co1—N3^i^	−124.2 (2)
N3—C28—C29—N4	0.7 (3)	C16—N1—Co1—O4^vi^	−147.6 (2)
N4—C27—N3—C28	0.3 (3)	C17—N1—Co1—O4^vi^	2.9 (3)
N4—C27—N3—Co1^i^	−177.40 (17)	C16—N1—Co1—O8	118.6 (2)
C29—C28—N3—C27	−0.6 (3)	C17—N1—Co1—O8	−90.9 (3)
C29—C28—N3—Co1^i^	176.76 (19)	C16—N1—Co1—O1	24.1 (2)
N3—C27—N4—C29	0.1 (3)	C17—N1—Co1—O1	174.6 (3)
N3—C27—N4—C26	−178.5 (3)	C16—N1—Co1—O7^iv^	−22.8 (7)
C28—C29—N4—C27	−0.4 (3)	C17—N1—Co1—O7^iv^	127.7 (5)
C28—C29—N4—C26	178.1 (3)	C16—N1—Co1—N3^i^	−63.4 (2)
C23—C26—N4—C27	115.6 (3)	C17—N1—Co1—N3^i^	87.1 (3)
C23—C26—N4—C29	−62.6 (4)	Co1—O8—Co2—O3^ii^	144.91 (9)
O4—C8—O3—Co2^ii^	−3.0 (4)	Co2^v^—O8—Co2—O3^ii^	−85.14 (12)
C6—C8—O3—Co2^ii^	176.27 (17)	Co1—O8—Co2—O6^iv^	−44.69 (10)
O3—C8—O4—Co1^iii^	−39.8 (4)	Co2^v^—O8—Co2—O6^iv^	85.26 (9)
C6—C8—O4—Co1^iii^	140.96 (19)	Co1—O8—Co2—O2	50.69 (9)
C10—C9—O5—C4	18.1 (4)	Co2^v^—O8—Co2—O2	−179.37 (7)
C14—C9—O5—C4	−163.6 (2)	Co1—O8—Co2—O8^v^	−129.95 (12)
C3—C4—O5—C9	−123.9 (3)	Co2^v^—O8—Co2—O8^v^	0.0
C5—C4—O5—C9	59.1 (3)	C1—O2—Co2—O3^ii^	−171.8 (2)
O7—C15—O6—Co2^iv^	27.6 (3)	C1—O2—Co2—O8	−31.0 (2)
C12—C15—O6—Co2^iv^	−151.95 (18)	C1—O2—Co2—O6^iv^	83.4 (2)
O6—C15—O7—Co1^iv^	−17.9 (5)	C1—O2—Co2—O8^v^	−48 (2)
C12—C15—O7—Co1^iv^	161.6 (2)		

Symmetry codes: (i) −*x*+2, −*y*+1, −*z*+1; (ii) −*x*+2, −*y*+2, −*z*; (iii) *x*+1, *y*+1, *z*; (iv) −*x*+2, −*y*+2, −*z*+1; (v) −*x*+1, −*y*+1, −*z*; (vi) *x*−1, *y*−1, *z*.
