# Supplementary figures and images for: Crystal structure of poly[[{μ2-1,4-bis[(1H-imid­azol-1-yl)methyl]benzene}[μ6-5-(4-carboxylatophenoxy)isophthalato]-μ3-hydroxido-dicobalt(II)] 0.25-hydrate]
Source: Acta Crystallogr Sect E Struct Rep Online. 2014 Oct 29;70(Pt 11):m376–7. doi: 10.1107/S1600536814022806 (PMC4257313; doi:10.1107/S1600536814022806)

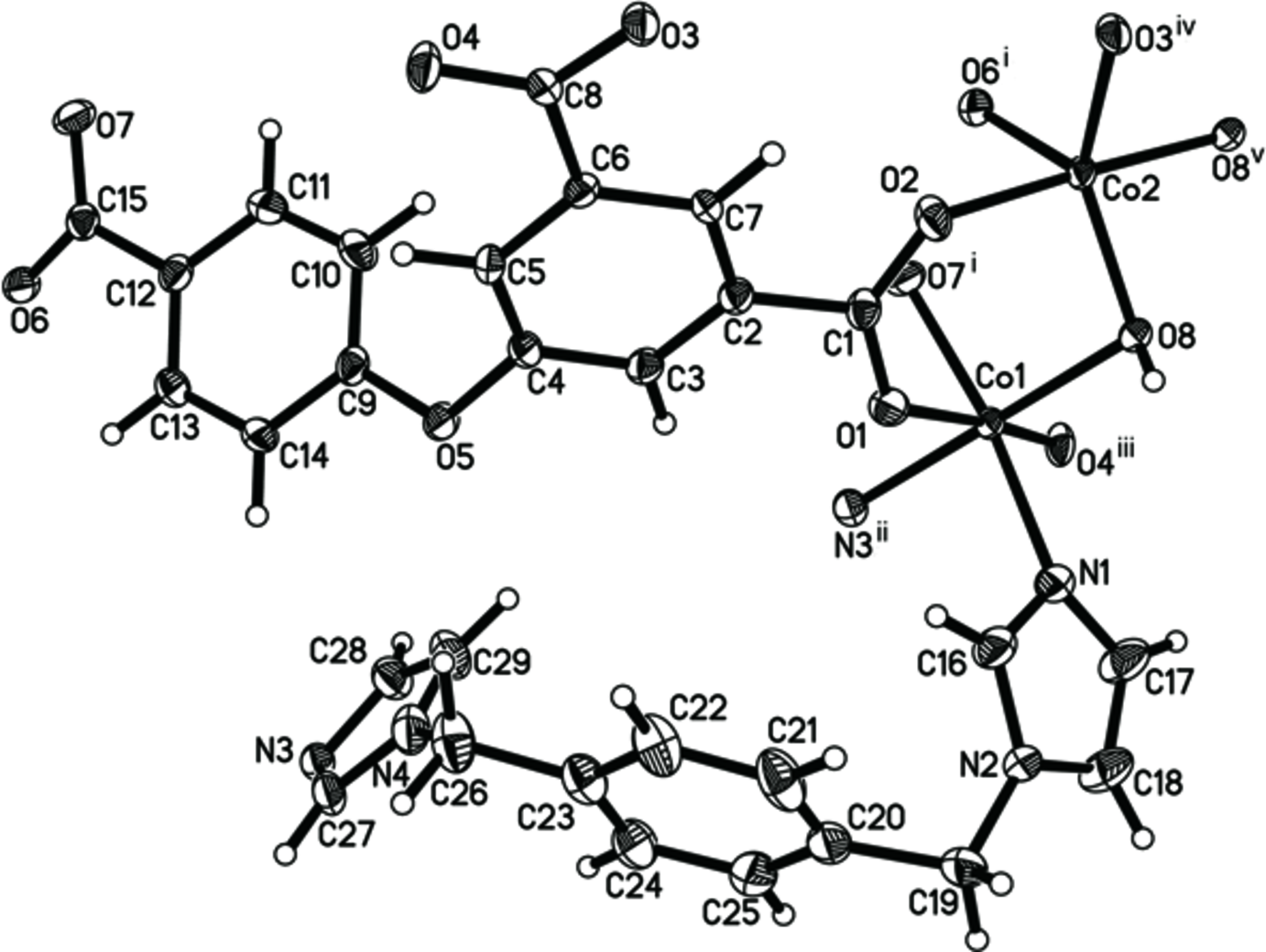

Supplement: Supplementary file 3 [file e-70-0m376-fig1.tif]

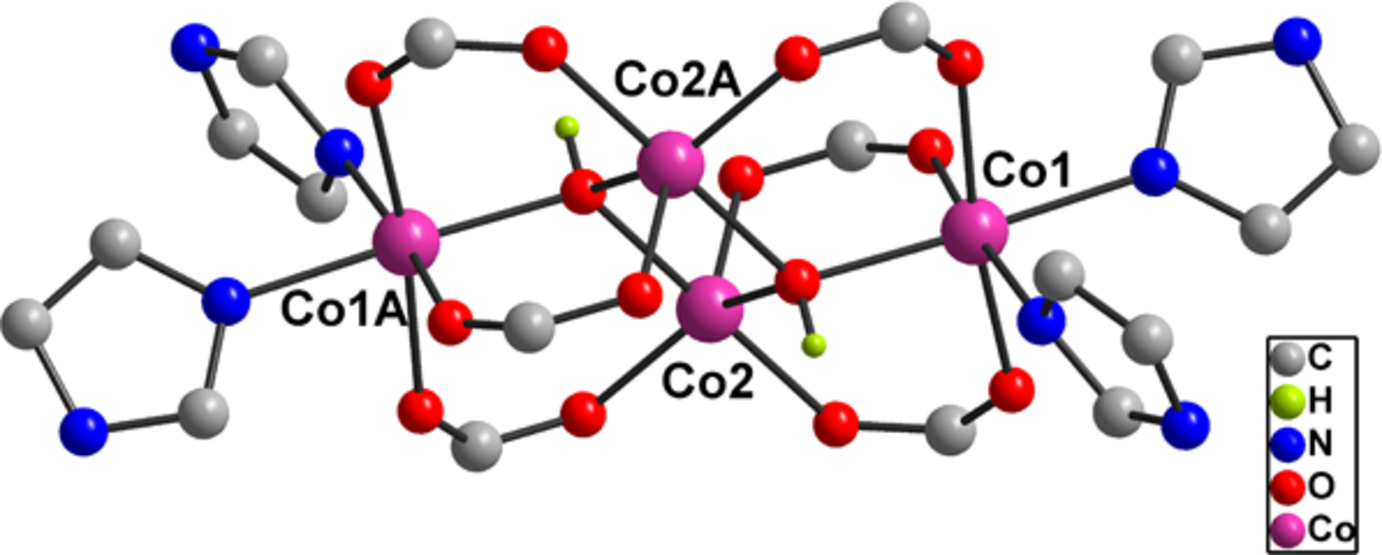

Supplement: Supplementary file 4 [file e-70-0m376-fig2.tif]

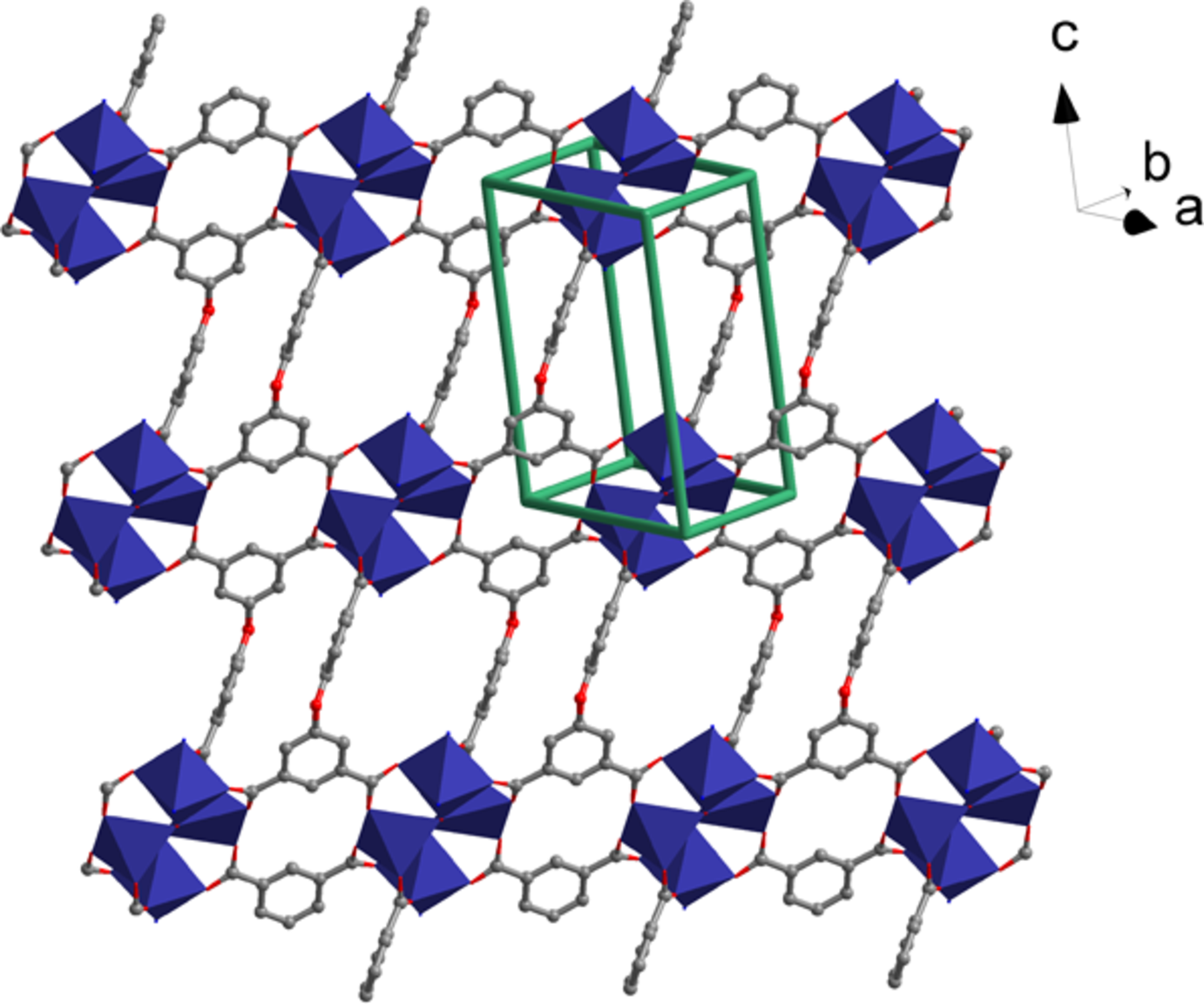

Supplement: Supplementary file 5 [file e-70-0m376-fig3.tif]
